# Epithelial/mesenchymal heterogeneity of high‐grade serous ovarian carcinoma samples correlates with miRNA let‐7 levels and predicts tumor growth and metastasis

**DOI:** 10.1002/1878-0261.12762

**Published:** 2020-08-21

**Authors:** Evgeny Chirshev, Nozomi Hojo, Antonella Bertucci, Linda Sanderman, Anthony Nguyen, Hanmin Wang, Tise Suzuki, Emmanuel Brito, Shannalee R. Martinez, Christine Castañón, Saied Mirshahidi, Marcelo E. Vazquez, Pamela Wat, Kerby C. Oberg, Yevgeniya J. Ioffe, Juli J. Unternaehrer

**Affiliations:** ^1^ Division of Biochemistry Department of Basic Sciences Loma Linda University Loma Linda CA USA; ^2^ Department of Pathology and Human Anatomy Loma Linda University School of Medicine Loma Linda CA USA; ^3^ Biology Department California State University San Bernardino San Bernardino CA USA; ^4^ Center for Health Disparities and Molecular Medicine Loma Linda University Loma Linda CA USA; ^5^ Biospecimen Laboratory Division of Microbiology & Molecular Genetics Department of Basic Sciences Loma Linda University Cancer Center Loma Linda University Loma Linda CA USA; ^6^ Department of Radiation Medicine Loma Linda University Loma Linda CA USA; ^7^ Division of Gynecologic Oncology Department of Gynecology and Obstetrics Loma Linda University Medical Center Loma Linda CA USA; ^8^ Department of Gynecology and Obstetrics Loma Linda University Loma Linda CA USA; ^9^ Present address: Laboratory for Prediction of Cell Systems Dynamics RIKEN Osaka 565‐0874 Japan

**Keywords:** epithelial/mesenchymal transition, high‐grade serous ovarian cancer, microRNA, orthotopic patient‐derived xenografts, stem cells, transcriptional regulation

## Abstract

Patient‐derived samples present an advantage over current cell line models of high‐grade serous ovarian cancer (HGSOC) that are not always reliable and phenotypically faithful models of *in vivo* HGSOC. To improve upon cell line models of HGSOC, we set out to characterize a panel of patient‐derived cells and determine their epithelial and mesenchymal characteristics. We analyzed RNA and protein expression levels in patient‐derived xenograft (PDX) models of HGSOC, and functionally characterized these models using flow cytometry, wound healing assays, invasion assays, and spheroid cultures. Besides *in vitro* work, we also evaluated the growth characteristics of PDX *in vivo* (orthotopic PDX). We found that all samples had hybrid characteristics, covering a spectrum from an epithelial‐to‐mesenchymal state. Samples with a stronger epithelial phenotype were more active in self‐renewal assays and more tumorigenic in orthotopic xenograft models as compared to samples with a stronger mesenchymal phenotype, which were more migratory and invasive. Additionally, we observed an inverse association between microRNA *let‐7* (*lethal‐7*) expression and stemness, consistent with the loss of *let‐7* being an important component of the cancer stem cell phenotype. We observed that lower *let‐7* levels were associated with the epithelial state and a lower epithelial mesenchymal transition (EMT) score, more efficient spheroid and tumor formation, and increased sensitivity to platinum‐based chemotherapy. Surprisingly, in these HGSOC cells, stemness could be dissociated from invasiveness: Cells with lower *let‐7* levels were more tumorigenic, but less migratory, and with a lower EMT score, than those with higher *let‐7* levels. We conclude that *let‐7* expression and epithelial/mesenchymal state are valuable predictors of HGSOC proliferation, *in vitro* self‐renewal, and tumor burden *in vivo*.

## Graphical abstract

We studied ovarian cancer patient‐derived cells to determine their epithelial vs. mesenchymal phenotype, and their stemness, migration, invasion, and tumor growth characteristics. Surprisingly, stemness could be dissociated from invasiveness. We observed that lower let‐7 levels are associated with the epithelial state and stemness, reliably predict self‐renewal and tumor burden in mice, and could contribute to prognosis calculations.

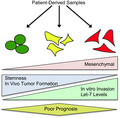

**Abbreviations**
AXL: receptor tyrosine kinase
CDH1: cadherin 1
CDH2: cadherin 2
CLDN3: claudin 3
CSC: cancer stem cell
EMT: epithelial/mesenchymal transition
FN1: fibronectin 1
FTSEC: fallopian tube secretory epithelial cells
GAS6: growth arrest‐specific 6
HGSOC: high‐grade serous ovarian carcinoma
HMGA2: high mobility group AT‐Hook 2
HR: homologous recombination
Let‐7: lethal‐7 microRNA
LIN28A: LIN28 homolog A
miR: microRNA
NANOG: homeobox transcription factor Nanog
NCCIT: pluripotent embryonal carcinoma cell line
NSG: NOD scid gamma
OCLN: occluding
PD: patient‐derived
PDX: patient‐derived xenograft
Rad51: DNA repair protein
SNAI1: snail 1 zinc finger protein
TGF‐b: transforming growth factor beta
TJP1: tight junction protein 1
UTR: untranslated region
BRCA1/2: DNA repair‐associated protein 1/2
OCT4 (POU5F1): octamer‐4 embryonic gene

## 1 Introduction

Advanced high‐grade ovarian cancer is the most lethal gynecological malignancy, with a 5‐year survival of approximately 30% [1]. High‐grade serous ovarian carcinoma (HGSOC) is the most common subtype and is responsible for the majority of patient deaths [2, 3]. Most (70–80%) of HGSOC patients respond well to front‐line treatment consisting of surgery and chemotherapy. However, 70–90% of patients relapse, resulting in poor subsequent prognosis and survival [4]. In the last decade, under the umbrella of HGSOC, several morphological and molecular tumor subtypes have been identified [3, 5, 6, 7]. Further classification of tumor heterogeneity will facilitate application of precision medicine techniques and is expected to improve treatment response rates and survival outcomes [8]. Targeted therapies are developed in an effort to minimize selection and proliferation of metastatic chemoresistant tumor clones [9]. Currently, in the HGSOC treatment paradigm, tumor recurrence after initial therapy is believed to occur, at least in part, due to the survival of a subpopulation of cancer stem cells (CSC), also known as tumor‐initiating cells within tumors, that evade initial surgery and chemotherapy and are responsible for maintenance and growth of metastatic tumors [10].

In order to move forward with the application of successful therapeutic approaches, development of better prognostic markers is needed [11]. In previous studies, the mesenchymal HGSOC molecular subtype (Mes, also called C1) was associated with higher platinum resistance, while the C5 (proliferative or Stem‐A subtype) has a dedifferentiated phenotype; both C1 and C5 subtypes have poor prognosis [6, 7, 12]. A subset of cancer cells has both epithelial and mesenchymal characteristics. This ‘hybrid’ phenotype is associated with CSC characteristics and poor prognosis [13, 14]. We and others showed that epithelial mesenchymal transition (EMT) inducer *SNAI1* (Snail) contributes to stemness, chemoresistance, and invasiveness of HGSOC [15, 16, 17]. Therefore, the presence of epithelial/mesenchymal phenotype can be used for tumor classification. Patients that present with certain mesenchymal and epithelial characteristics may benefit from a more personalized course of treatment and more frequent follow‐up. This leads to the prediction that close surveillance of individuals at risk for cancer relapse and metastasis, predicted by EMT status, can result in timely diagnosis and treatment.

In addition to epithelial/mesenchymal status, analysis of cellular microRNAs (miRNA) has been used to stratify tumors and predict response to therapy [18, 19]. miRNAs are small (~ 22 nucleotides) noncoding RNAs that regulate gene expression post‐transcriptionally by binding the 3′ UTR [20]. miRNAs have been shown to be abnormally expressed in HGSOC, affecting cisplatin‐induced apoptosis [21, 22, 23, 24]. *Let‐7* miRNA was first discovered to regulate development in *Caenorhabditis elegans* and is conserved across species [25]. *Let‐7* is important in regulation of stem cell differentiation in both worms and mammals, and its repression in adult stem cells increases ability to self‐renew [26, 27, 28]. *Let‐7* targets a large group of oncogenes, pluripotency factors, cell cycle regulators, and components of DNA damage repair pathways [29]. Effects of *let‐7* dysregulation are thus dependent on the set of target genes expressed in a particular cell. *Let‐7* is required for maintaining the differentiated state of somatic cells, and must be repressed for reprogramming somatic cells to pluripotency [30]. *Let‐7* is repressed in several cancers, including ovarian, and its decrease is associated with an increase in stemness, resulting in poor prognosis [21, 31, 32, 33, 34, 35]. The associations between *let‐*7 and ovarian cancer prognosis point to *let‐7*'s potential for use as a biomarker [32]. Due to the differential expression of *let‐7* targets, we hypothesize that *let‐7* expression can be used to classify HGSOC for prediction of tumor characteristics.

Here, we characterized patient‐derived HGSOC cells with regard to epithelial and mesenchymal characteristics, stemness and chemoresistance phenotype, *let‐7* levels, and the ability to form tumors in immunocompromised mice. We show that while *let‐7* levels correlated indirectly, as expected, with stemness and *in vivo* growth, surprisingly *let‐7* levels directly associated with other measures of aggressiveness, including migratory ability and invasiveness. We also confirm in patient‐derived cells that samples with both epithelial and mesenchymal features were associated with higher self‐renewal ability, chemoresistance, and tumor formation in orthotopic xenografts, as we previously demonstrated in cell lines [15]. These findings demonstrate the possibility of categorizing and predicting HGSOC phenotype and resistance based on epithelial/mesenchymal phenotype and *let‐7* levels.

## 2 Methods

### 2.1 Cell culture

All studies were approved by the Loma Linda University (LLU) Institutional Review Board (IRB, 58238) and performed according to the standards of the Declaration of Helsinki. Tumor tissues from ovarian cancer patients were collected by Loma Linda University Cancer Center Biospecimen Laboratory (LLUCCBL), after informed consent. The same tumor samples were used to clinically diagnose HGSOC. Deidentified fresh ovarian cancer samples were processed by mincing and passing over a 100‐μm strainer using the plunger of a 3‐mL syringe. Some fibrous samples were digested with Dispase (StemCell Technologies, Vancouver, BC, Canada) for 4–16 h. After pelleting, erythrocytes were removed by overlaying a cell suspension on a 3 mL Ficoll gradient. Cells were engrafted into NSG (NOD.Cg‐*Prkdc^scid^ Il2rg^tm1Wjl^*/SzJ, Jackson Labs, Bar Harbor, ME, USA, stock #005557) mice subcutaneously in the region of the mammary fat pad, resulting in patient‐derived xenografts (PDX) after 3–14 months. Cancer cell enrichment was not done. Of 55 HGSOC samples, 14 engrafted or expanded sufficiently for *in vitro* use. Of these, eight had *in vitro* growth characteristics conducive to the experiments carried out here. The entire set of eight is included here. Patient‐derived samples were cultured in three‐part Ham's F12 and one‐part Dulbecco's modified Eagle's medium (DMEM) (all media from Fisher Scientific, Pittsburgh, PA, USA), supplemented with 5% FBS (Omega Scientific, Tarzana, CA, USA), 10 µm insulin (all chemicals from Sigma‐Aldrich, St. Louis, MO, USA, unless otherwise stated), 0.4 μm hydrocortisone, 2 μg·mL^−1^ isoprenaline, 24 μg·mL^−1^ adenine, 100 U·mL^−1^ of penicillin, and 10 μg·mL^−1^ streptomycin (Fisher Scientific). 5–10 μm Y27632 (Fisher Scientific) was added to establish growth *in vitro* [36]. Low passage patient‐derived cells (maximum 15) were used to avoid changes induced by extensive passaging in *in vitro* culture.

OVCAR8 human ovarian cancer cell line was a gift from Carlotta Glackin (City of Hope), fallopian tube secretory epithelial cells (FTSEC) from Ronny Drapkin, and NCCIT embryonal carcinoma cell line from George Daley (Harvard Medical School). OVCAR8 cells were cultured in DMEM with 10% FBS, 2 mm l‐Glutamine (Fisher Scientific), 100 U·mL^−1^ penicillin, and 10 μg·mL^−1^ streptomycin. NCCIT cells were cultured in RPMI medium with 10% FBS, 2 mm l‐Glutamine, 1% nonessential amino acids, 1 mm sodium pyruvate, 100 U·mL^−1^ penicillin, and 10 μg·mL^−1^ streptomycin. FTSEC were cultured in DMEM–Ham's F12 50/50 supplemented with 2% USG (Crescent Chemical, Islandia, NY, USA), 100 U·mL^−1^ penicillin, and 10 µg·mL^−1^ streptomycin [37].

### 2.2 Real‐time quantitative reverse transcription–PCR

Total RNA from cell culture samples was isolated using TRIzol reagent (Life Technologies, Carlsbad, CA, USA) according to the manufacturer's instructions. For mRNA expression analysis, cDNA was synthesized with 1 μg of total RNA using Maxima First Strand cDNA Synthesis Kit (K1672; Thermo Fisher Scientific, Grand Island, NY, USA). Real time reverse transcriptase quantitative PCR (RT‐qPCR) for mRNA was performed using PowerUP SYBR Green Master Mix (Thermo Fisher Scientific) and specific primers on a Stratagene Mx3005P Instrument (Agilent Technology, Santa Clara, CA, USA). The sequence of primers for mRNA quantitation is shown in Table S1. For miRNA expression analysis, cDNA was synthesized with 100 ng of total RNA using specific stem–loop RT primers and TaqMan microRNA Reverse Transcription Kit (Applied Biosystems, Foster City, CA, USA). Real‐time RT‐qPCR for miRNA was performed using TaqMan Universal PCR Master Mix II (Applied Biosystems) with TaqMan probes (Life Technologies) on a Stratagene Mx3005P Instrument (Agilent Technology). The primers and probes for miRNA quantitation were supplied with the TaqMan microRNA Assay (Applied Biosystems). The results were analyzed using the ΔΔ cycles to threshold (ΔΔ*C*_t_) method.

### 2.3 Spheroid formation assay

Cells were plated at a density of 1000 cells·mL^−1^ in nontissue culture‐coated plates (Olympus plastics, Genessee Scientific, San Diego, CA, USA) and maintained for 7 days in serum‐free medium (DMEM/F12 50/50) supplemented with 0.4% bovine serum albumin, 10 ng·mL^−1^ FGF, 20 ng·mL^−1^ EGF, 6.7 ng·mL^−1^ selenium, 5.5 µg·mL^−1^ transferrin, 10 µg·mL^−1^ insulin, and 1% knockout serum replacement (Gibco, Thermo Fisher Scientific). Seven days later, the number of spheroids was counted and statistically analyzed. Phase‐contrast images of spheroids were taken and analyzed using imagej software (National Institutes of Health, Bethesda, MD, USA) to assess the size of spheroids.

### 2.4 Scratch assay (wound healing cell migration assay)

Cells were grown to 90% confluency in 24‐well tissue culture plates, then treated with mitomycin C and scratched with a 10‐μL pipet tip. Pictures of fixed positions in the wounds were taken every 4 h for a 24‐h period with a brightfield Nikon Eclipse Ti microscope (Nikon Instruments, Melville, NY, USA) with phase contrast. The wound area in each picture was measured by imagej software (National Institutes of Health, Bethesda, MD, USA).

### 2.5 Western blot

Cells were lysed in Laemmli buffer and sonicated, and proteins were separated by SDS/PAGE and transferred to a PVDF membrane. To prevent nonspecific binding, the membrane was blocked with 0.1%‐5% milk in Tris‐buffered saline with 0.2% Tween (TBST) for 1 h for each immunoblotting antibody. Immunoblotting was performed with primary antibodies including α/β‐tubulin, LIN28A, and HMGA2 (Cell Signaling Technology, Danvers, MA, USA). Secondary antibody immunoblotting was done with anti‐rabbit IgG conjugated with DyLight 680 (Invitrogen, Carlsbad, CA, USA). PVDF membranes were imaged by Odyssey LI‐COR Infrared Imaging System (LI‐COR Biosciences, Lincoln, NE, USA). Densitometry analysis was performed by ImageJ software (National Institutes of Health, Bethesda, MD, USA).

### 2.6 Cell viability assay

3‐(4,5‐dimethylthiazol‐2‐yl)‐2,5‐diphenyltetrazolium bromide (MTT; Sigma‐Aldrich) assays were used to determine cell viability. Cells were seeded at a density of 1000 cells/well in 96‐well plates and incubated overnight. The cells were then treated with increasing concentrations of cisplatin, paclitaxel, and olaparib for 72 h. After drug treatment, MTT solution was added to each well, and the plates were incubated for 2 h at 37 °C. The formed formazan crystals were dissolved in dimethyl sulfoxide, and the absorbance was measured at 570 nm using a SpectraMax i3x microplate reader (Molecular Devices, Sunnyvale, CA, USA). The half‐maximal inhibitory concentration (IC50) of drug was analyzed using the graphpad prism version 7.0 (GraphPad Software, La Jolla, CA, USA).

### 2.7 Mice

All experiments were performed in accordance with the NIH guidelines for the humane care and use of laboratory animals, and were approved by the Institutional Animal Care and Use Committee at Loma Linda University. Nude (J:NU) and NOD *scid* gamma (NSG) mice obtained from the Jackson Laboratory (Bar Harbor, ME, USA) were housed in specific pathogen‐free conditions, and were used for xenografts at 6–10 weeks of age.

### 2.8 Orthotopic xenograft model

Under isoflurane anesthesia, PDX3, PDX9, PDX5, PDX6, PDX8, and PDX4 cells were injected into the ovarian bursa of nude or NSG mice at 2.5 × 10^5^ cells per mouse (PDX3 *n* = 3, PDX5 *n* = 7, PDX9 *n* = 4, PDX6 *n* = 7, PDX8 *n* = 4, PDX6 *n* = 11, and PDX4 *n* = 6) in PBS, mixed 1 : 1 with Matrigel (354248; Corning, Corning, NY, USA). Weight and girth were measured biweekly to assess general health and tumor burden, and mice were euthanized at humane endpoints, or a maximum of 90 days.

### 2.9 Rad51/ionizing radiation induced *ex vivo* foci assay

Homologous recombination status was determined after ionizing radiation (IR) induced DNA damage, by assessing Rad51 activation. Cells were plated onto a glass slide and exposed to ionizing radiation (protons). Cell irradiation was performed at room temperature using a 250 MeV proton beam as previously described [38]. Samples were exposed to a proton dose of 4 Gy at the center of the spread‐out Bragg peak (SOBP) or mock‐irradiated. Thirty minutes and 8 h after irradiation, optimal time for ɣH2AX and Rad51 focus formation, cells were rinsed with PBS and then fixed with ice‐cold methanol at room temperature for 15 min [39, 40]. Cells were then permeabilized with 0.5% Triton X‐100 in PBS for 5 min at room temperature and washed three times in PBS. Nonspecific antigens were blocked for 30 min in 5% goat serum in 0.3% Triton X‐100 at room temperature. Samples were incubated with primary antibodies, against ɣH2AX (ab26350; Abcam, Cambridge, MA, USA) and Rad51 (ab68301; Abcam), in 5% goat serum and 0.3% Triton X‐100 for 1 h at room temperature, then washed five times in PBS and then incubated for 1 h at room temperature with Alexa Fluor 488‐ and Alexa Fluor 568‐conjugated secondary antibodies (A‐11029; A‐11031; Life Technologies). After six washes with PBS, cells were DAPI‐counterstained and mounted with ProLong Diamond Antifade Mountant (Life Technologies). All cells were analyzed and imaged using a Nikon Eclipse Ti microscope and micromanager software (US National Institute of Health).

### 2.10 Flow cytometry

Cells were placed in FACS stain (PBS with 1% FBS, 0.1% sodium azide, and 2 mm EDTA) and labeled with conjugated fluorescent dye antibodies against CD44 (130‐110‐298), CD117 (130‐099‐325), CD133 (130‐090‐854), CD324 (E‐Cadherin) (130‐099‐141) obtained from Miltenyi Biotec (Auburn, CA, USA), and CD325 (N‐Cadherin) (350814; BioLegend, San Diego, CA, USA). Cells were then incubated for 15 min in the dark at 4 °C. After incubation, cells were washed and fixed with FACS Fix (FACS stain with 1% PFA). UltraComp eBeads (01‐2222; Thermo Fisher Scientific) were used for compensation. Flow cytometry was performed on MACS Quant Analyzer 10 (Miltenyi Biotec), and analysis of data was performed using flowjo 10 (FlowJo LLC, Ashland, OR, USA).

### 2.11 Morphological measurements and EMT score calculation

Aspect ratio was calculated using imagej. Individual cells were manually outlined using freehand selection tool, and aspect ratio was calculated by measuring length and width; aspect ratio = width/length. To obtain an accurate aspect ratio for each sample, three biological replicates (*n* = 3), with minimum of 20 cells per replicate, were photographed and analyzed by several individuals to avoid bias. We calculated EMT score by using RT‐qPCR levels of three epithelial (*OCLN, TJP1,* and *CLDN3*) and three mesenchymal (*SNAI1, CDH2,* and *FN1*) markers selected on the basis of the minimal number of factors able to predict E vs. M status in agreement with morphological characteristics. We modified the formula used by Chae *et al*. [41], substituting *z* score with expression of RNA markers relative to housekeeping gene β‐actin and subtracting epithelial markers from mesenchymal markers [41]: EMT score = Mean of mesenchymal markers − mean of epithelial markers. Human BJ1 fibroblasts and MCF10A samples served as mesenchymal and epithelial controls, respectively.

### 2.12 Invasion assay

Prior to assay, cells were cultured for 24 h in their respective media without FBS. Transwell inserts were coated with 0.1 μg basement membrane extract (BME) (3433‐010‐01) in 1× Cultrex coating buffer (3455‐096‐03) (Trevigen, Gaithersburg, MD, USA) and placed in incubator for 24 h. On the day of the assay, cells were dissociated from culture dish with 0.05% Trypsin/EDTA, and counted and resuspended in serum‐free media. 20 000 cells (PDX4 and PDX6) and 50 000 cells (PDX3, PDX5, PDX9, PDX8, and OVCAR8) in 200 μL of serum‐free media were plated on inserts. Cell number was optimized for proliferation rate. Media containing FBS was added to the bottom chamber. Cells were placed in a 37 °C incubator for 24 h. The top of inserts was wiped clean with a cotton‐tipped applicator; then, inserts were fixed in 70% ethanol for 15 min, and stained in 0.2% crystal violet for 10–15 min and rinsed with dH_2_O. Once dry, cells on bottom of insert were imaged at a total magnification of 100× using Leica DMi1 inverted microscope (Leica Microsystems, Boulder Grove, IL, USA) and counted using imagej. Samples in which 50 000 cells were plated onto insert were normalized to 20 000.

### 2.13 Statistical analysis

Graphical figures and statistical analysis were performed using graphpad prism version 7.0 (GraphPad Software). Detailed information on statistical analysis is described in figure legends.

## 3 Results

### 3.1 Epithelial/mesenchymal phenotype of HGSOC cells

**Fig. 1:**
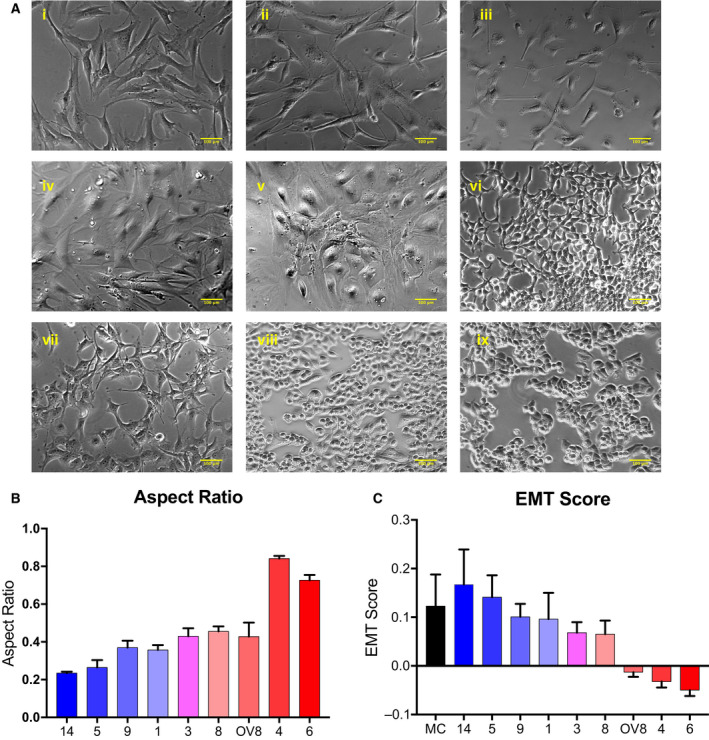
Epithelial/mesenchymal characterization of HGSOC cells. (A) Patient‐derived samples and OVCAR8 cell line (A, i–viii; i = PDX14, ii = PDX5, iii = PDX9, iv = PDX1, v = PDX3, vi = PDX8, vii = OVCAR8, viii = PDX4, ix = PDX6). Scale bar represents 100 μm. The passage numbers at which images were taken are 6, 9, 5, 3, 8, 8, 8, 9, and 11, respectively. Morphological analysis (B) was performed on three independent biological replicates (*n* = 3), where 1 = epithelial. (C) EMT score was calculated by RNA expression of mesenchymal and epithelial markers (three each; see Methods), demonstrates negative correlation with morphology and aspect ratio. MC, BJ1 fibroblast as mesenchymal control; EC, MCF10A as epithelial control. Independent biological replicates (*n*): PDX14 = 5, PDX5 = 4, PDX9 = 4, PDX1 = 5, PDX3 = 3, PDX8 = 4, OV8 = 4, PDX4 = 4, PDX6 = 4.

Cancer stem cells are often described as having not only stemness qualities, but also invasiveness [42]. We hypothesized that patient‐derived (PD) samples with enhanced stemness would also be more migratory and invasive. Here, we characterized PD HGSOC cells, retaining all tumor cells to accurately assess tumor heterogeneity. We refer to them as PDX after they have successfully engrafted in NSG mice (see Methods). We evaluated eight PD HGSOC samples (PDX14, PDX5, PDX9, PDX1, PDX3, PDX8, PDX6, and PDX4). Table 1 provides relevant clinical information about the samples. Histology is shown in Fig. S1. We analyzed cell morphology in phase‐contrast micrographs (Fig. 1A), comparing patient‐derived cells with OVCAR8, one of the HGSOC cell lines we previously analyzed for its epithelial and mesenchymal characteristics [15], whose gene expression profile closely resembles HGSOC [43]. HGSOC cells were characterized based on epithelial/mesenchymal phenotype. We quantified these traits with aspect ratio measurements, shown to correlate with epithelial and mesenchymal phenotype (Fig. 1B). Epithelial cells are generally more circular (with an aspect ratio closer to one) because they are tightly opposed to one another in a cobblestone‐like pattern, while mesenchymal cells are more elongated [44]. Epithelial cells have a ratio of 1 that indicates cobblestone shape, while mesenchymal cells have a lower ratio [44, 45, 46]. We used RNA expression of three epithelial markers (*OCLN, TJP1,* and *CLDN3*) and three mesenchymal markers (*SNAI1, CDH2,* and *FN1*) to calculate the EMT score [41] (see Methods), where samples with more mesenchymal phenotype have a positive score while more epithelial samples are negative (Fig. 1C). Human BJ1 fibroblast and MCF10A were used as mesenchymal and epithelial controls, respectively. The EMT score negatively correlates with cell morphology calculated by aspect ratio with the Spearman correlation of −0.8871 (*P* = 0.0014) (Fig. 1B). Images of cells in Fig. 1 are arranged left to right from high to low EMT score. We observed that all samples contained cells expressing both CDH1 and CDH2, as well as a population of cells positive for both markers (Fig. S2). Expression of EMT inducer Snail (Snai1) on the protein level demonstrates that all samples, including those with most epithelial features, express mesenchymal characteristics (Fig. S3). Thus, all samples contained cells exhibiting partial EMT or hybrid phenotype, but could be placed along an EMT spectrum based on morphological, gene, and protein expression criteria.

**Table 1:** Clinical, pathological, and functional characteristics of HGSOC patient‐derived samples. PFS, progression‐free survival. c/t, carboplatin/paclitaxel.

	PDX	Stage	Source	Chemotherapy	PFS (months)
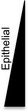	14	IVA	Ovary	4 cycles c/t	No relapse
	5	IVB	Ovary, cul‐de‐sac	No prior chemotherapy	18
	9	IIA	Omentum	4 cycles c/t	10
	1	IVB	Ascites	No prior chemotherapy	15
	3	IVB	Ovary	6 cycles c/t	36
	8	IIIC	Tumor	6 cycles c/t	7.5
	4	IVB	Ovary, omentum	No prior chemotherapy	16
	6	IIIC	Ascites	No prior chemotherapy	No relapse

### 3.2 HGSOC division rate, migration, and invasion

**Fig. 2:**
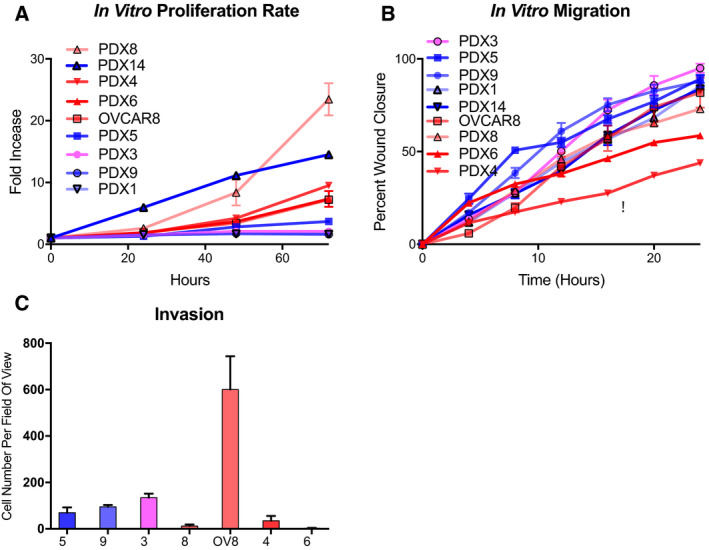
Proliferation, migration, and invasion of HGSOC cells. Doubling time (A), migration (B), and invasion assay (C) performed via MTT, wound healing assay, and Matrigel invasion assay, respectively. Independent biological replicates; PDX5 (*n* = 5), PDX9 (*n* = 3), PDX1 (*n* = 4), PDX3 (*n* = 3), PDX8 (*n* = 3), OV8 (*n* = 3), PDX4 (*n* = 3), and PDX6 (*n* = 3). Migration is represented in technical replicates that have been performed in biological triplicates. Invasion is represented by independent biological replicates (*n* = 3). Samples are labeled from top to bottom in order of decreasing growth rate and migration. OV8 = OVCAR8 cell line. Error bars represent standard error of the mean (SEM).

Population doubling time was measured with MTT assays; cells with lower EMT score cycled more rapidly (Fig. 2A). Migratory ability of cells was assessed with wound healing assays. As expected, cells with higher EMT score were more migratory, with the Spearman correlation of 0.7223 (*P* = 0.028) (Fig. 2B). Ability of cells to invade was assayed via Matrigel invasion assay (Fig. 2C).

### 3.3 HGSOC stemness and self‐renewal

**Fig. 3:**
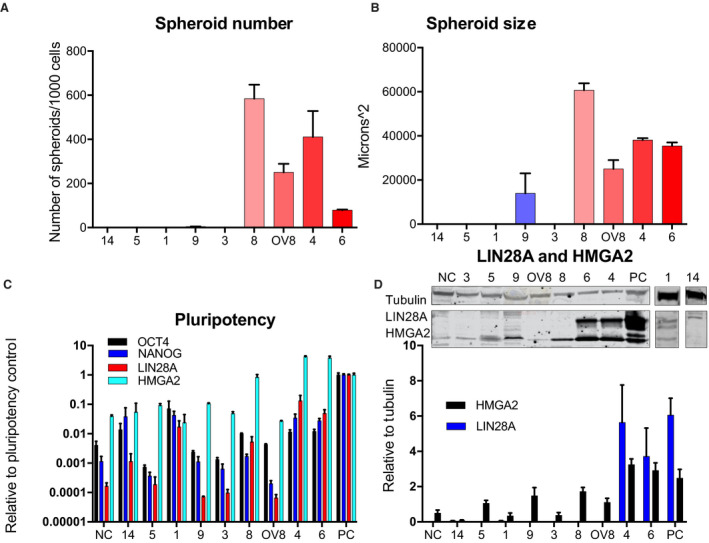
HGSOC pluripotency and self‐renewal. Cells were grown in nonadherent conditions, and spheroids were analyzed on day 7. Spheroid number per 1000 cells (A) and size (B) (PDX9 (*n* = 5), PDX8 (*n* = 5), OV8 (*n* = 5), PDX4 (*n* = 5), and PDX6 (*n* = 3); error bars, SEM). PDX14, PDX5, PDX1, and PDX3 did not form spheroids. Pluripotency markers OCT4, NANOG, LIN28A, and HMGA2 were analyzed by RT‐qPCR (C). (D) Western blots of LIN28A and HMGA2 relative to tubulin were quantified on protein level. Full western blots shown in Fig. S5. LIN28A was not detected in PDX3, 5, 9, 8, and OV8.

We used spheroid formation assays to demonstrate self‐renewal ability, a stem cell feature [15, 47]. PDX cells with lower EMT score formed more and larger spheroids compared to samples with higher EMT score (Fig. 3A,B, Fig. S4). To understand self‐renewal of PDX samples, pluripotency was assessed by detecting both RNA and protein levels. RNA expression of pluripotency markers OCT4 (*POU5F1)*, *NANOG, LIN28A,* and *HMGA2* is shown in Fig. 3C. FTSEC [37] and human embryonal carcinoma (NCCIT) samples served as normal (NC) and pluripotency (PC) controls, respectively. The cells with lower EMT score expressed high levels of these markers. Protein levels confirmed that cells with lower EMT score express higher levels of *LIN28A* and *HMGA2* as shown in Fig. 3D and Fig. S5. EMT score negatively correlates with *LIN28A* and *HMGA2* protein levels with the Spearman correlation of −0.7132 (*P* = 0.031) and −0.7703 (*P* = 0.0151), respectively. CD117 and CD133 are well‐established ovarian CSC markers [48]. Our previous studies are consistent with this population being enriched in CSC [15]. We analyzed expression of these CSC markers (Figs [Link], [Link]); no correlation between these markers and EMT score was observed. Taken together, cells with lower EMT score had more stem cell characteristics.

### 3.4 *Let‐7* family members are repressed in HGSOC

**Fig. 4:**
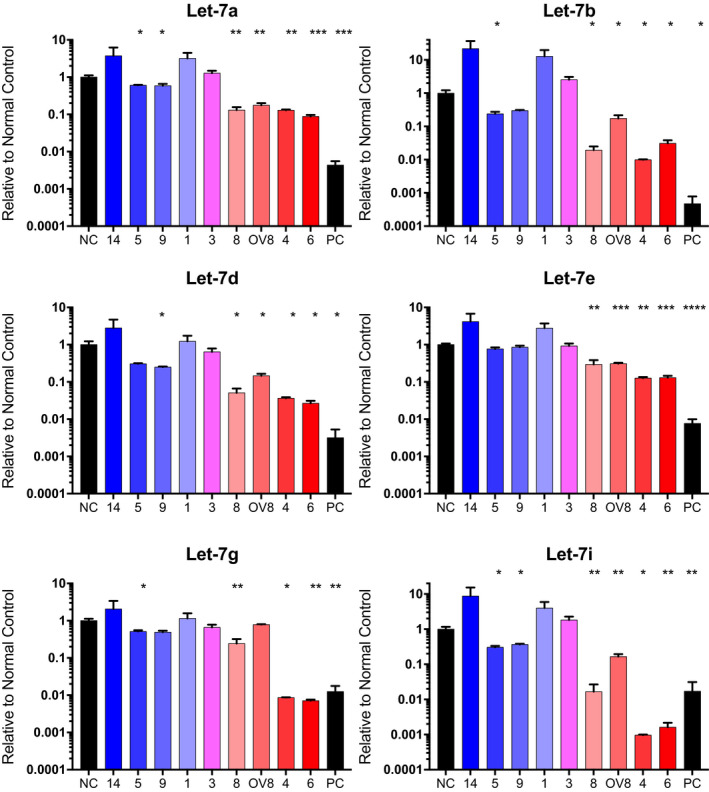
Let‐7 is repressed in HGSOC. RT‐qPCR of let‐7a, b, d, e, g, and i in PDX samples and OVCAR8 compared to normal control. Independent replicates *n*; NC (5), PDX14 (3), PDX5 (3), PDX9 (3), PDX1 (3), PDX3 (4), PDX8 (3), OV8 (3), PDX4 (3), PDX6 (3), PC (3). Significance was calculated via Student's *t*‐test with *P ≤ 0.05, **P ≤ 0.01, ***P ≤ 0.001, and ****P ≤ 0.0001. Error bars: SEM.

Our previous data suggested that the EMT transcription factor Snail increases stemness directly via *let‐7* [15]. Snail represses *let‐7* levels transcriptionally during reprogramming; therefore, similar mechanisms are likely to repress *let‐7* in HGSOC cells as well [30] (H. Wang, E. Chirshev, N. Hojo, T. Suzuki, A. Bertucci, C. Perry, R. Wang, J. Zink, C. A. Glackin, Y. J. Ioffe and J.J. Unternaehrer, unpublished data). Higher levels of pluripotency markers and self‐renewal ability suggest the presence of CSCs. MicroRNA let‐7 has been shown to be essential for somatic cells to maintain the differentiated state, and its loss leads to acquisition of stemness and carcinogenesis. Higher levels of *let‐7* have been shown to be indicative of more differentiated cancer cells [31]. Tumor‐suppressive actions of *let‐7* are attributed to its role in suppressing oncogenes and genes involved in maintenance of pluripotency, such as HMGA2 and LIN28A [49], and *let‐7* directly targets these genes in ovarian cancer [31]. Therefore, we evaluated levels of six *let‐7* family members in HGSOC patient samples. Levels of most *let‐7* family members were observed to be repressed in all HGSOC samples, with lowest *let‐7* levels in samples with low EMT score (Fig. 4). Student's *t*‐test was used to calculate significance compared to normal control. In this group of samples, loss of *let‐7* associated with epithelial features. *Let‐7* was negatively correlated with aspect ratio, RNA levels of pluripotency marker *HMGA2*, proliferation, invasion, spheroid number and size, and *in vivo* tumor growth, formation, and burden. Table S2 shows the Spearman correlation coefficients and significance.

### 3.5 Chemoresistance of HGSOC cells

We determined resistance to the commonly used chemotherapy agent cisplatin (Table 2, Fig. S8) by treatment with increasing concentrations of drug. Sensitivity to cisplatin is partially attributable to homologous recombination (HR) status. Mutation in several genes in the pathway can result in HR deficiency; one of these is *BRCA2*, mutation of which increases ovarian cancer cell sensitivity to platinum‐based therapy [50]. PDX4 and PDX6 are BRCA2 mutant, while PDX5 is BRCA1 mutant, as determined by clinical testing (see Table 2); OVCAR8 is wild‐type, and genetic testing of PDX 8 was not done clinically. We determined HR repair status of all samples by adapting an established assay to qualitatively observe Rad51 foci formation [51] (Table 2). HR‐deficient cells fail to form Rad51 foci in response to DNA damage [52]. For our testing, DNA damage was induced by ionizing radiation. In concordance with the genetic testing results, PDX9 and PDX3 were found to be HR‐proficient, while PDX4, PDX6, and PDX5 were deficient (Table 2). PDX8 was determined to be HR‐deficient. We observed that PDX samples with higher EMT score were associated with higher resistance compared to those with lower EMT score and had significant correlation with *let‐7* levels (Table S2). However, because the low *let‐7*/low EMT score samples were HR‐deficient, while many of the high *let‐7/*high EMT score samples were HR‐competent, sample size was insufficient to stratify groups based on both EMT score and HR status, and we are unable to determine whether resistance is attributable to EMT status, *let‐7* levels, HR status, or a combination.

**Table 2:** Chemoresistance, BRCA status, and homologous recombination (HR) status of HGSOC samples. IC50 values of cisplatin, determined by MTT viability assay. Genetic testing of samples was performed during clinical diagnosis. HR status was confirmed via foci formation assay (see Methods).

	Sample	Cisplatin (μm)	BRCA status	HR status
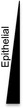	PDX14	13	WT	Deficient
	PDX5	4.1	BRCA1 mutation	Proficient
	PDX9	5.8	WT	Proficient
	PDX1	25	WT	Deficient
	PDX3	6.0	WT	Proficient
	PDX8	1.5	Unknown	Deficient
	OVCAR8	6.8	WT^a^	Proficient
	PDX4	0.7	BRCA2 mutation	Deficient
	PDX6	0.4	BRCA2 mutation	Deficient

^a^ Methylated [89].

### 3.6 HGSOC tumor growth and metastasis *in vivo*

To test the ability of PDX cells to grow *in vivo*, cells were injected into the ovarian bursae of Nude or NOD scid gamma (NSG) mice. Primary and metastatic tumor formation (at maximum 90 days), days to endpoint, and size of primary tumors were interrogated (Table 3). Tumorigenesis represents percentage of xenografts that formed tumors at the site of primary orthotopic injection into ovarian bursa. Samples with lower *let‐7* levels and higher aspect ratio (Fig. 1B) positively correlated with primary tumor size at the time of necropsy with the Spearman correlation coefficient of 0.88 (*P* = 0.05). These samples with lower *let‐7* levels, high aspect ratio, and lower EMT score also reached endpoint (defined as a 25% increase in abdominal girth) in > 90% of cases. Samples with a higher *let‐7* expression and higher EMT score did not form observable tumors at the primary site of injection within 90 days. Thus, it was samples with low *let‐7* levels and low EMT score that were most tumorigenic and low tumor *let‐7* levels can be used to predict rapid growth and metastasis.

**Table 3:** PDX *in vivo* model. Nude mice injected orthotopically with PDX. Tumorigenicity refers to percentage of tumors formed at the primary site of injection (ovarian bursa) at euthanasia, maximum 90 days. Metastasis refers to percentage of mice that developed secondary tumors. Numbers of mice orthotopically injected: *N* = 3, 4, 7, 4, 11, and 6 for PDX3, PDX9, PDX5, PDX8, PDX6, and PDX4 respectively. NSG, NOD *scid* gamma.

	Sample	Strain	Tumorigenicity (%)	Endpoint reached (%)	Endpoint (days)	Primary tumor (g)	Metastasis (%)	Notes
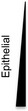	PDX5	NSG	0	0	NA	NA	0	
	PDX9	Nude	0	0	NA	NA	0	
	PDX3	Nude/NSG	0	0	NA	NA	0	
	PDX8	NSG^c^	100	100	36.3 ± 1.7	6.3 ± 0.34	100	a
	PDX4	Nude	100	100	44.7 ± 4.7	9.24 ± 0.95	40	b
	PDX6	Nude	90	90	23.7 ± 2.6	5.4 ± 1.2	73	b

^a^ Bloody ascites. Peritoneal and liver metastasis. Primary tumor invaded peritoneum and was difficult to excise.

^b^ Peritoneal metastases.

^c^ No tumors formed in nude mice.

## 4 Discussion

We characterized a panel of eight primary tumor samples in two‐ and three‐dimensional *in vitro* cultures and in orthotopic PDX, focusing on their epithelial/mesenchymal and stemness properties, as well as their aggressiveness, which we define as the ability to migrate, invade, self‐renew, and form tumors *in vivo*, and their chemoresistance. PD samples with lower *let‐7* levels were most active in assays for self‐renewal (Fig. 3) and tumorigenicity (Table 3), and were more epithelial in nature. In our cell migration and invasion studies, we observed that the patient‐derived cells with higher *let‐7* levels were most invasive (Fig. 2), while those with higher EMT score were most migratory (Fig. 2), as expected. Surprisingly, samples that by these criteria were more mesenchymal had less stem cell‐like characteristics. In this system, we could thus dissociate stemness from invasiveness, in apparent contrast to findings in ovarian cancer cell lines [15, 53]. The limitations of this study are the small number of samples and the relatively small numbers of genes examined. Our objective in these studies was to characterize these samples deeply on both phenotypic and functional levels, and we chose depth over breadth to this end. We aimed to develop a measure of the epithelial/mesenchymal status of a tumor sample that would be accessible when genomic profiling was not available.

It should be noted that in the cell lines we and others previously examined, a narrower range of phenotypes on the epithelial/mesenchymal spectrum was observed: All cell lines were found to be hybrid [15]; none would have been characterized as fully mesenchymal or epithelial. While OVCAR8 (EMT score −0.013) was the most mesenchymal of the cell lines in our previous publication, several of our patient‐derived cells had higher EMT scores than OVCAR8 (Fig. 2). Similarly, in our previous study, OVSAHO was the most epithelial line, and its EMT score is −0.017, while two patient‐derived samples had scores well below zero (−0.032, −0.050), and were thus further toward the epithelial end of the spectrum. Thus, the spectrum of patient‐derived cells is broader than that of cell lines. We previously demonstrated that, of cell lines examined, OVCAR8 had the highest percentage of surface stemness markers and was also most aggressive of the cell lines examined in terms of migration, invasion, and self‐renewal ability [15]. In the patient‐derived samples, the more mesenchymal cells were more invasive and migratory, but they were less active in assays for stemness. The cells with the most robust growth characteristics in 3D culture were cells with lower *let‐7* levels (Fig. 3).

In our panel of patient‐derived cells, those with lower *let‐7* levels and lower EMT scores were associated with higher sensitive to cisplatin (Table 2). However, this may be due to their known status as *BRCA2* mutants (from patients' clinical data). BRCA1 represses EMT via transcriptional effects on Twist [54, 55]; no association between *BRCA2* and EMT has been drawn. Our patient samples with no known mutations are more resistant to cisplatin, as expected [50]. Further testing of additional samples is necessary to draw conclusions whether sensitivity is more closely associated with epithelial/mesenchymal status, or with HR status. Interestingly, we did not observe that samples from patients previously treated with chemotherapy (carboplatin and taxol) were consistently more cisplatin‐resistant than the samples from chemotherapy‐naïve patients (Tables 1 and 2).

Growth of patient‐derived cells in orthotopic PDX was assessed after ovarian bursa injection. Cells that formed rapidly growing primary tumors in these models were those with lower *let‐7* levels and lower EMT score. Of the subsets that were robustly tumorigenic, it was the sample with moderate EMT score (PDX8) that demonstrated most invasive growth, forming metastases and bloody ascites. Similar to PDX8, OVCAR8 has previously been shown to be highly tumorigenic, producing peritoneal metastasis and ascites [56]. We conclude that cells retaining epithelial characteristics are more tumorigenic *in vivo*.

While in solid tumors EMT has been widely linked with acquisition of stemness [57, 58, 59], closer examination has revealed that partial rather than full EMT is associated with stemness [60]. EMT can occur to varying extents; partial EMTs resulting in hybrid states are associated with acquisition of stem cell characteristics [61, 62, 63, 64]. The presence of both mesenchymal and epithelial characteristics we observed in PDX 8, 6, and 4 may thus contribute to their enhanced stemness in terms of self‐renewal and tumorigenicity. Samples that fall toward the mesenchymal end of the spectrum, PDX14, 5, 9, 1, 3, display less epithelial characteristics in terms of morphology, and less self‐renewal and tumorigenicity. It is possible that the samples with higher *let‐7* levels and higher EMT score have gained invasiveness at the expense of stemness. The relationship between EMT and tumor progression in ovarian cancer is complex [14, 65, 66], but our findings, which are in agreement with others who have reported on epithelial plasticity as it relates to ovarian CSCs [14, 66, 67, 68, 69], add to the evidence for partial EMT cells, those that retain epithelial characteristics, being more active in terms of proliferation, self‐renewal, and tumorigenicity.

Because of its key role in the differentiated cell status, we assessed *let‐7* levels in the patient‐derived samples. We observed that patient‐derived cells with higher levels of stemness markers (*LIN28A, OCT4, NANOG*; Fig. 3) also had lower *let‐7* levels (Fig. 4). There were also correlations between *let‐7* levels and doubling time, and indirect associations with spheroid formation, *in vivo* tumor burden, and metastasis. The Spearman correlation coefficients and significance are given in Table S2. The ability to divide rapidly in samples with low *let‐7* is presumed to be at least partially due to *let‐7* targeting genes involved in cell cycle progression [49]. On the basis of our data, in all samples examined loss of all *let‐7* family members correlates with markers of stemness and tumorigenicity. While EMT score associates with some characteristics of PD samples, *let‐7* levels serve as an even better predictor for *in vitro* aggressiveness and *in vivo* metastasis. Although most reports are consistent with all members of the *let‐7* family playing tumor‐suppressive roles in ovarian cancer [31, 32, 70, 71], caution is warranted because *miR‐98* and *let‐7b* have also been shown to be associated with poor outcome [72, 73]. Further studies are necessary to fully understand the roles of specific *let‐7* family members.

Previous studies have begun to elucidate the basis of differences between molecular subtypes of ovarian cancer. The C1 or mesenchymal (Mes) subtype is associated with desmoplasia, activation of the TGF‐b pathway, miRNA dysregulation, and worse outcome [6, 7, 74, 75]. In the C5 or proliferative (Stem‐A) subtype, in addition to expression of genes related to proliferation, extracellular matrix‐related genes are highly expressed, and *let‐7* deregulation is specifically associated with this subtype, driven by *MYCN amplification* [6, 76]. Both of these subsets express mesenchymal markers [6]. The most strongly overexpressed gene in C5 cells is HMGA2 [76]; in our study, cells with low *let‐7* levels and low EMT score expressed high levels of HMGA2 on both mRNA and protein levels (Fig. 3C,D). *Let‐7* levels can predict HGMA2 expression due to directly targeting HMGA2 mRNA. While we did not formally assess molecular subtype of samples, the low *let‐7*/low EMT score samples could equate to the C5/proliferative/Stem‐A subtype: Despite their low EMT score, they express high levels of Snail; they express low levels of *let‐*7; they are highly proliferative; and they express HMGA2. Those with high *let‐7* levels/high EMT score are possibly C1/Mes, with lower expression of epithelial genes, lower rates of proliferation, and more mesenchymal characteristics in terms of migration and invasion [67, 77]. Studies addressing molecular subtype have not yet determined how function varies with different gene expression signatures. Some studies have assessed molecular subtype in cell lines [7, 78, 79] and provided insights into phenotype of cells of known subtype. One study assessed cell lines of C1/Mes vs. C5/proliferative subtypes for functional characteristics, focusing on inhibiting TGF‐b signaling via the GAS6/AXL pathway [79]. In this study, differences in response were observed in cell lines of the two subtypes tested, demonstrating that differences between subtypes can be leveraged for precision medicine approaches. These studies provide the basis for more work delineating subtype‐specific therapies.

Cell lines have formed the basis for cancer knowledge, but their limitations have become more evident with time. Although cell lines have provided the basis for many descriptive, mechanistic, and preclinical cancer studies, their limitations are widely recognized [80, 81, 82, 83]. The NCI made the decision to halt the use of its panel of 60 cancer cell lines, the NCI‐60, for drug screening in favor of patient‐derived samples [84]. To adequately represent the diversity present in HGSOC, characterization of patient‐derived cells is necessary [85, 86, 87]. Freshly obtained patient‐derived samples present several advantages over the use of cell lines: They maintain intratumoral and intertumoral heterogeneity, and available clinical data can add to the depth of knowledge about cells used [81, 82]. Primary tumor cultures are a valuable tool for preclinical studies: They provide subtle molecular and phenotypic diversity as evidenced in PDX, and thus a better platform for characterizing cancer than cell lines [87, 88].

This study provides evidence that information about *let‐7* levels and epithelial/mesenchymal phenotype may prove useful prognostically. Low EMT score samples uniformly expressed low levels of the tumor suppressor miRNA *let‐7*. The negative correlation of *let‐7* levels with tumorigenicity points to its potential for use as predictor of recurrence risk. Low EMT score can predict low *let‐7* levels of PD samples. Together with genetic testing, *let‐7* levels and EMT score could contribute to prognosis calculations, informing the decision for appropriate monitoring and course of treatment. Thus, low *let‐7* levels/low EMT score show potential as biomarkers for recurrence risk, while high *let‐7* level/high EMT score is a promising predictor of invasiveness and metastasis.

## 5 Conclusions

We found that the spectrum of variation is much broader in patient‐derived cells than in HGSOC cell lines. We devised an EMT scoring method to describe cells based on a six‐gene expression panel that corresponds to morphological assessments. Surprisingly, in these HGSOC cells, the patient samples with highest evidence of stemness were not the most invasive samples. Cells with lowest *let‐7* levels were less invasive, but more tumorigenic, and were on the lower spectrum of the EMT score than those with higher *let‐7* levels. We conclude that assessment of *let‐7* levels and epithelial/mesenchymal gene expression are viable candidates as predictors of HGSOC aggressiveness.

## Conflict of interest

The authors declare no conflict of interest.

## Author contributions

EC contributed to conceptualization, methodology, validation, formal analysis, investigation, writing of original draft, review and editing, visualization, and project administration. NH contributed to methodology, validation, and investigation. AB contributed to methodology, validation, investigation, and writing of original draft. LS, AN, HW, and TS contributed to methodology and investigation. EB, CC, and PW performed investigation. SM performed investigation, formal analysis, and contributed to resources. MEV contributed to methodology, validation, and investigation. KCO performed investigation and wrote, reviewed, and edited the manuscript. YJI contributed to conceptualization, methodology, formal analysis, investigation, writing of original draft, and reviewing and editing of the manuscript. JJU contributed to conceptualization, methodology, validation, formal analysis, investigation, writing of original draft, reviewing and editing, visualization, supervision, project administration, and funding acquisition. SRM performed investigation and formal analysis. SM contributed to resources.

## Supporting information


**Fig. S1.** The original tumor in PDX5 was a high‐grade papillary serous carcinoma. The tumor cells contained large vesicular nuclei with prominent nucleoli. The papillary character of the tumor is absent when grown in the mouse, but the cytologic features persist. The original tumors associated with PDX8 was diagnosed as high‐grade serous carcinoma and was composed of an admixture of smaller highly proliferative cells with monochromatic nuclei and larger tumor cells with more vesicular nuclei. In the mouse, the smaller tumor cells, consistent with the original, predominate with a high proliferative rate (numerous mitoses are evident). The patient's tumor associated with PDX4 was diagnosed as high‐grade serous carcinoma and displayed clusters of tumor cells in somewhat of an endometrioid pattern. These structural features did not translate to the mouse, but the cytology was similar with a high proliferative rate and mitotic figures. The PDX6's original tumor was diagnosed as poorly differentiated serous carcinoma, and within the mouse the cytology persisted with vesicular nuclei and prominent nucleoli. The images were taken at the same magnification (10x) and a 100 um scale bar is included in the left lower image.

**Fig. S2.** Flow cytometry of CDH1 and CDH2 in patient‐derived samples. Co‐expression of CDH1 and CDH2 via flow cytometry demonstrates both epithelial and mesenchymal characteristics of HGSOC PD samples. Independent biological replicates (n = 3), error bars = SEM.

**Fig. S3.** Snail expression in patient‐derived cells. A. WB of PDX (numbers indicated); NC: normal control (fallopian tube secretory epithelial cells); OV8: OVCAR8; PC: pluripotency control (NCCIT). Snail expression on protein level relative to TUBULIN demonstrates mesenchymal characteristics of all HGSOC patient‐derived samples and OVCAR8. B. Quantification of Snail expression at protein level from biological replicates. N = 3; error bars: SEM.

**Fig. S4.** Spheroids formed by patient‐derived samples at 10x magnification. i = PDX9, ii = PDX8, iii = OV8, iv = PDX4, v = PDX6. Scale bar: 100 μm.

**Fig. S5.** Full western blot membranes demonstrating position of human TUBULIN (55kD), Snail (29kD), LIN28A (26kD), and HMGA2 (18kD) in PDX samples along with OVCAR8, normal control (NC), and pluripotency control (PC). Protein ladder (left) demonstrates position of each band. Upper blot: PDX as indicated (3, 5, 9, 8, 6, 4); lower panels: PDX1 (left); PDX 14 (right). Relates to Fig. 3D, Fig. S1.

**Fig. S6.** Flow cytometry of (A) CD117+, (B) CD133+, and (C) CD117+/CD133+ population in patient‐derived parental cells and spheroid cells. Bars: SEM. n: 3 independent biological replicates for parental samples of PDX 14, 5, 1, 9, 3, 8, 4, 6, and OVCAR8, 3 for spheroid samples of PDX 8 and 4, and 5 for spheroid sample of PDX 6.

**Fig. S7.** Flow cytometry histograms. In all panels, isotype is shown in red, antibody in blue. PDX numbers are shown to the left of histograms. Antibodies are as shown in column headings.

**Fig. S8.** Cisplatin log curves demonstrate resistance in PD samples. Dashed lines represent 95% CI. Samples are arranged top to bottom in order of decreasing resistance. Independent biological replicates (n): PDX14 = 3, PDX5 = 7, PDX9 = 5, PDX1 = 3, PDX3 = 5, PDX8 = 4, OV8 = 4, PDX4 = 5, PDX6 = 5.

**Table S1.** RT‐qPCR human primer sequences
**Table S2.** Correlation between *let‐7* levels and patient‐derived sample phenotypic and functional characteristics.

## Data Availability

All data are available and will be collaboratively shared upon reasonable request.
