# Supplementary figures and images for: Epithelial/mesenchymal heterogeneity of high‐grade serous ovarian carcinoma samples correlates with miRNA let‐7 levels and predicts tumor growth and metastasis
Source: Mol Oncol. 2020 Aug 21;14(11):2796–813. doi: 10.1002/1878-0261.12762 (PMC7607177; doi:10.1002/1878-0261.12762)

**Supplementary Figure S1**

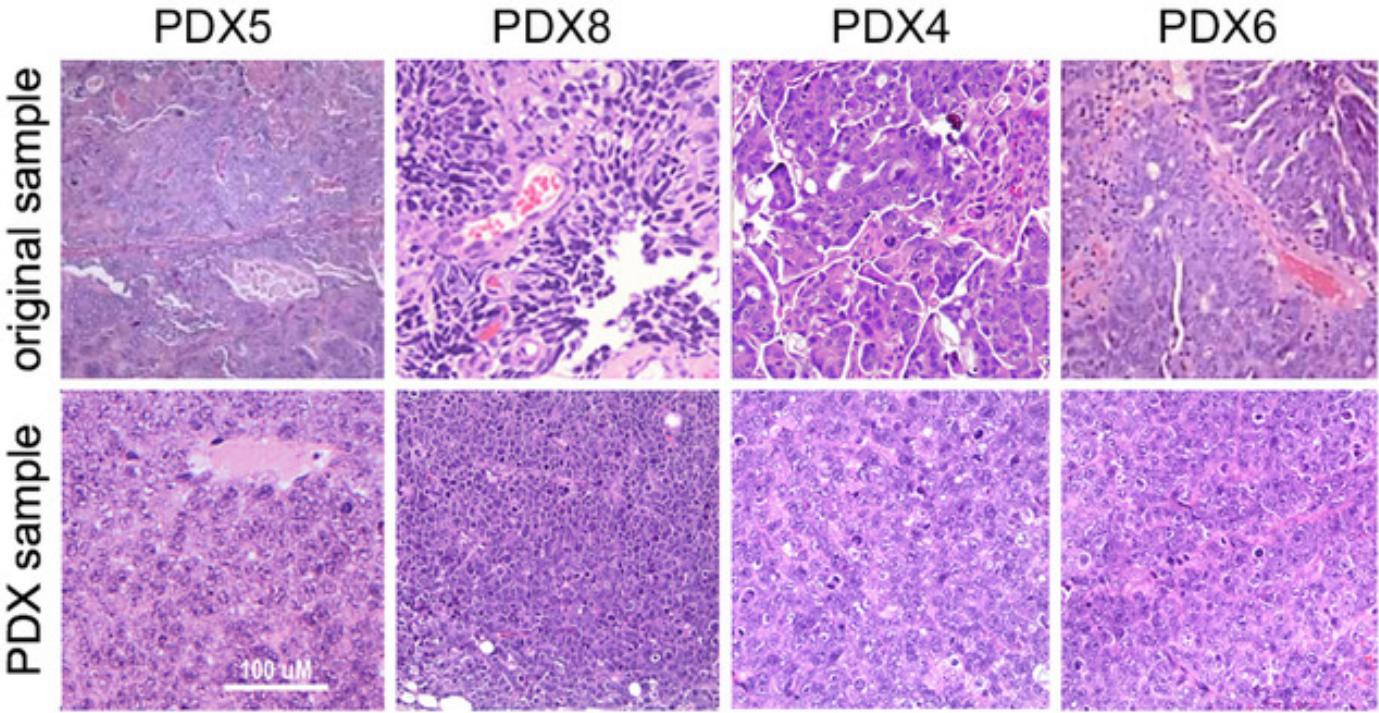

Supplement: Supplementary file 1 — Fig. S1. The original tumor in PDX5 was a high‐grade papillary serous carcinoma. The tumor cells contained large vesicular nuclei with prominent nucleoli. The papillary character of the tumor is absent when grown in the mouse, but the cytologic features persist. The original tumors associated with PDX8 was diagnosed as high‐grade serous carcinoma and was composed of an admixture of smaller highly proliferative cells with monochromatic nuclei and larger tumor cells with more vesicular nuclei. In the mouse, the smaller tumor cells, consistent with the original, predominate with a high proliferative rate (numerous mitoses are evident). The patient's tumor associated with PDX4 was diagnosed as high‐grade serous carcinoma and displayed clusters of tumor cells in somewhat of an endometrioid pattern. These structural features did not translate to the mouse, but the cytology was similar with a high proliferative rate and mitotic figures. The PDX6's original tumor was diagnosed as poorly differentiated serous carcinoma, and within the mouse the cytology persisted with vesicular nuclei and prominent nucleoli. The images were taken at the same magnification (10x) and a 100 um scale bar is included in the left lower image. [file MOL2-14-2796-s001.pdf]

## Supplementary Figure S2

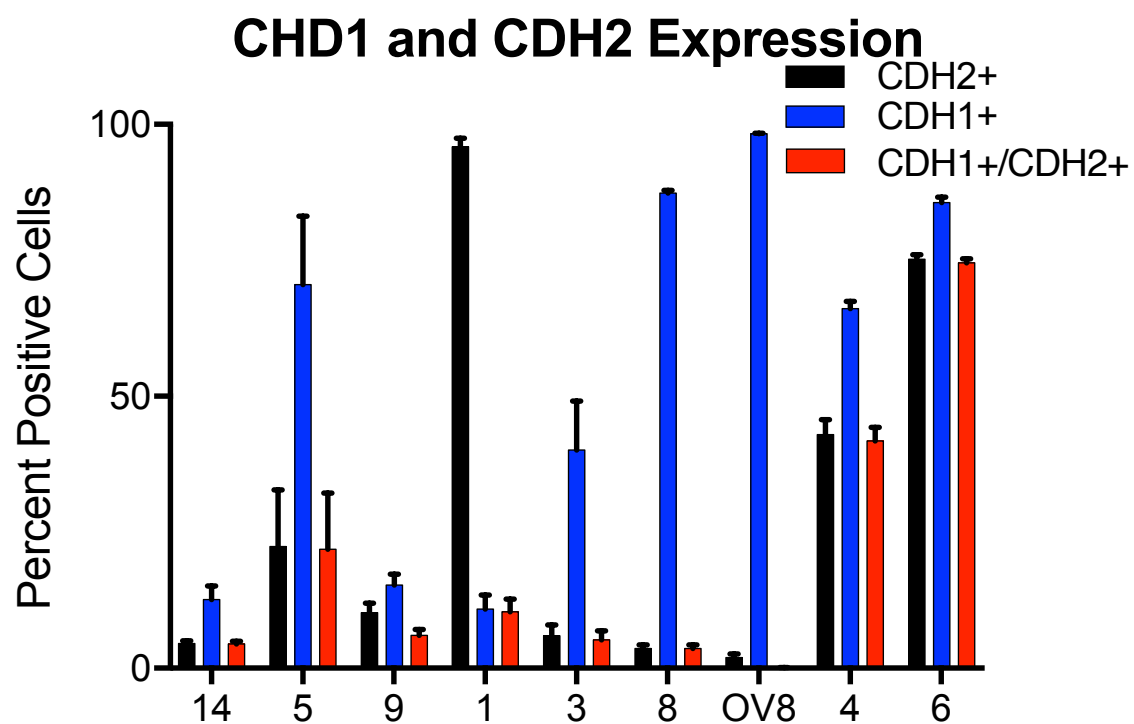

Supplement: Supplementary file 2 — Fig. S2. Flow cytometry of CDH1 and CDH2 in patient‐derived samples. Co‐expression of CDH1 and CDH2 via flow cytometry demonstrates both epithelial and mesenchymal characteristics of HGSOC PD samples. Independent biological replicates (n = 3), error bars = SEM. [file MOL2-14-2796-s002.pdf]

# Supplementary Figure S3

**A**

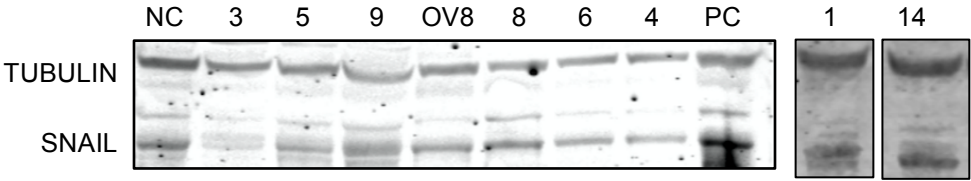

**B**

## Snail Expression

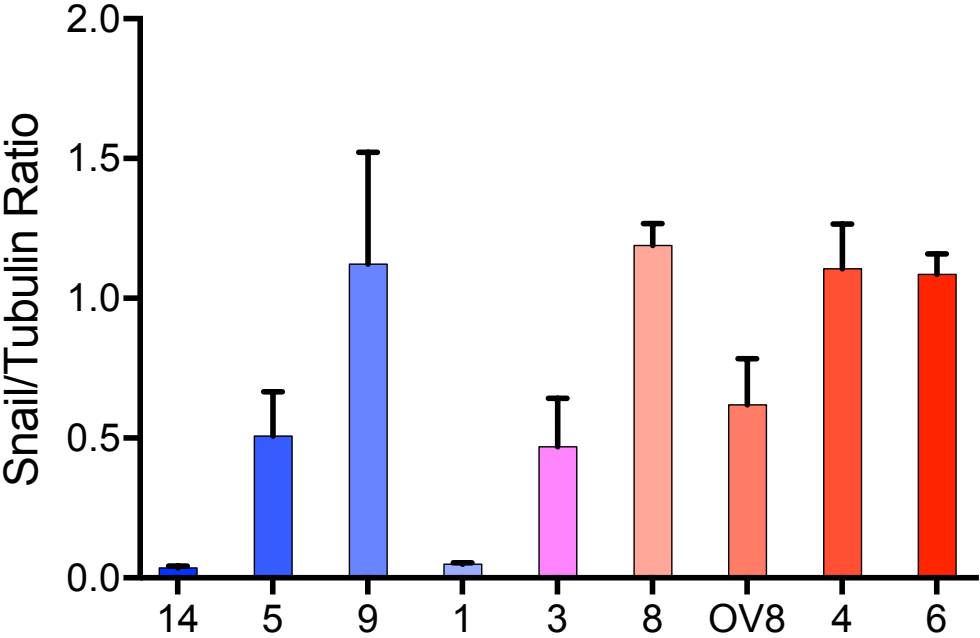

Supplement: Supplementary file 3 — Fig. S3. Snail expression in patient‐derived cells. A. WB of PDX (numbers indicated); NC: normal control (fallopian tube secretory epithelial cells); OV8: OVCAR8; PC: pluripotency control (NCCIT). Snail expression on protein level relative to TUBULIN demonstrates mesenchymal characteristics of all HGSOC patient‐derived samples and OVCAR8. B. Quantification of Snail expression at protein level from biological replicates. N = 3; error bars: SEM. [file MOL2-14-2796-s003.pdf]

## Supplementary Figure S4

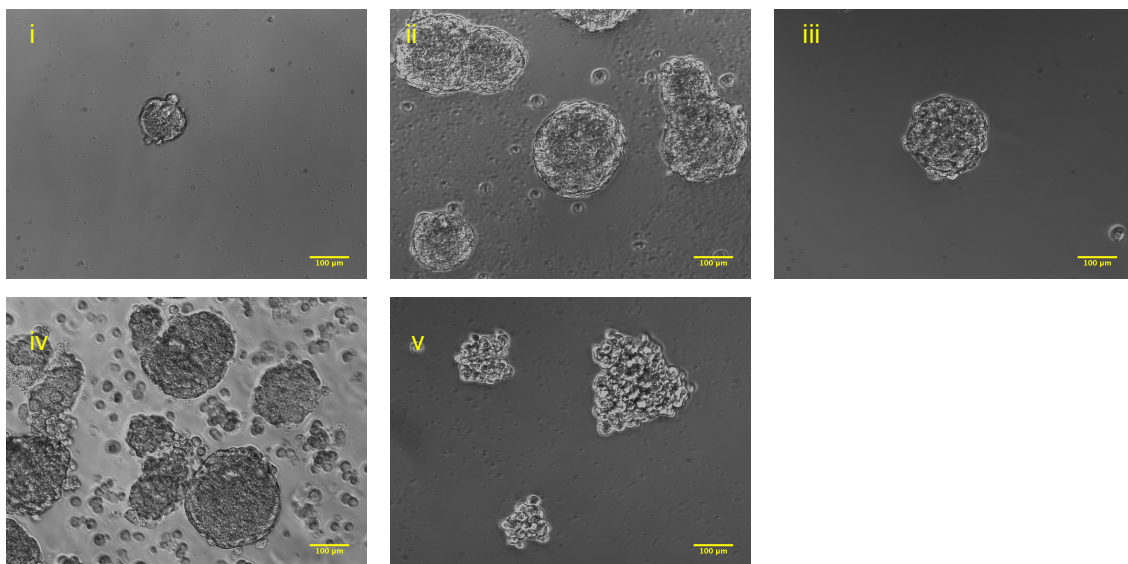

Supplement: Supplementary file 4 — Fig. S4. Spheroids formed by patient‐derived samples at 10x magnification. i = PDX9, ii = PDX8, iii = OV8, iv = PDX4, v = PDX6. Scale bar: 100 μm. [file MOL2-14-2796-s004.pdf]

# Supplementary Figure S5

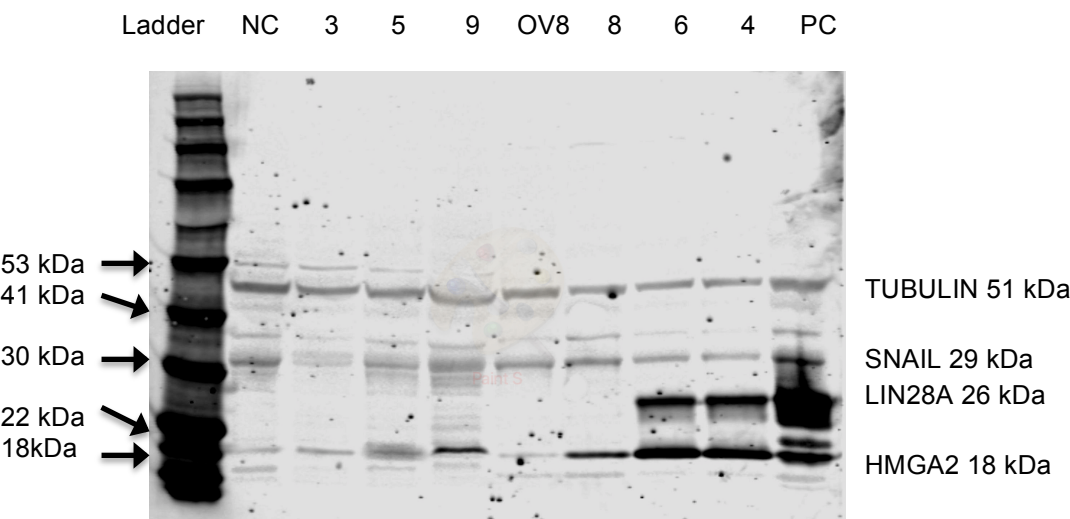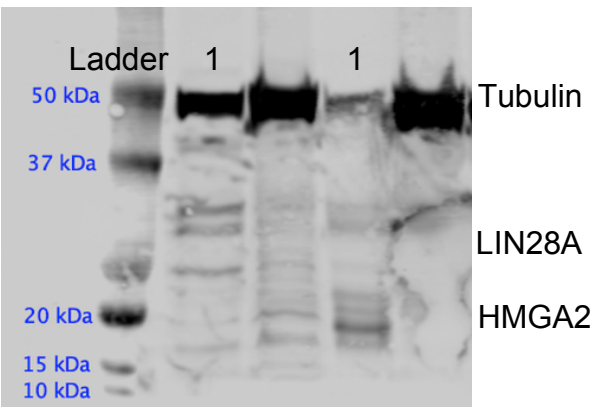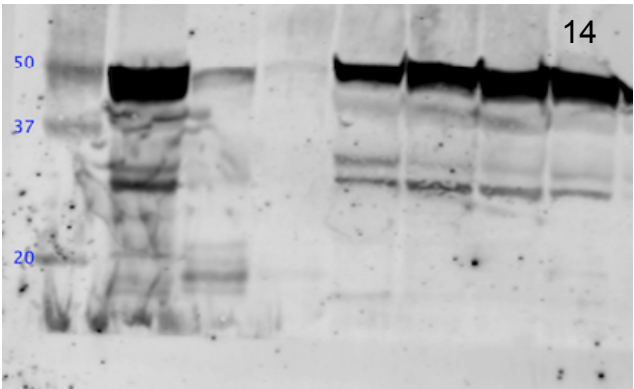

Supplement: Supplementary file 5 — Fig. S5. Full western blot membranes demonstrating position of human TUBULIN (55kD), Snail (29kD), LIN28A (26kD), and HMGA2 (18kD) in PDX samples along with OVCAR8, normal control (NC), and pluripotency control (PC). Protein ladder (left) demonstrates position of each band. Upper blot: PDX as indicated (3, 5, 9, 8, 6, 4); lower panels: PDX1 (left); PDX 14 (right). Relates to Fig. 3D, Fig. S1. [file MOL2-14-2796-s005.pdf]

# Supplementary Figure S6

A

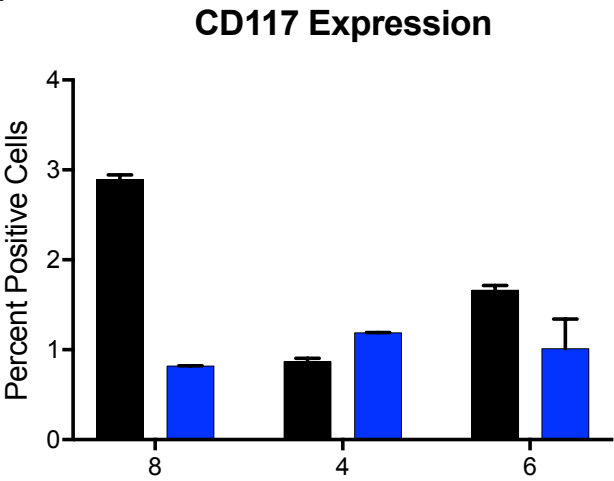

B

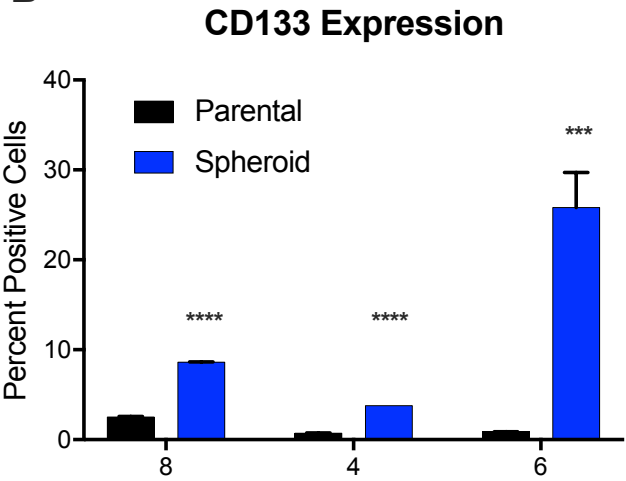

C

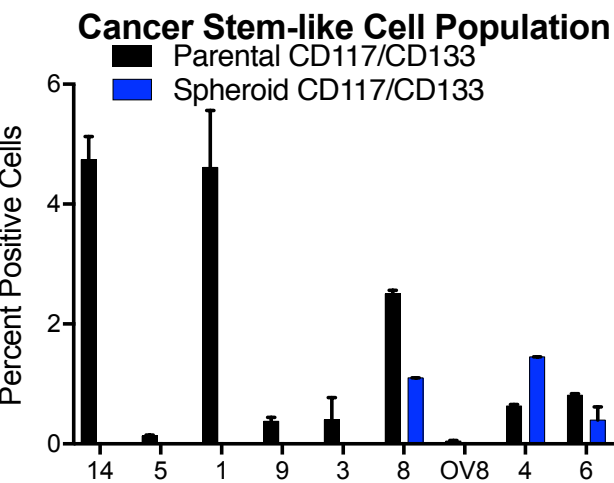

Supplement: Supplementary file 6 — Fig. S6. Flow cytometry of (A) CD117+, (B) CD133+, and (C) CD117+/CD133+ population in patient‐derived parental cells and spheroid cells. Bars: SEM. n: 3 independent biological replicates for parental samples of PDX 14, 5, 1, 9, 3, 8, 4, 6, and OVCAR8, 3 for spheroid samples of PDX 8 and 4, and 5 for spheroid sample of PDX 6. [file MOL2-14-2796-s006.pdf]

Supplementary Figure S7

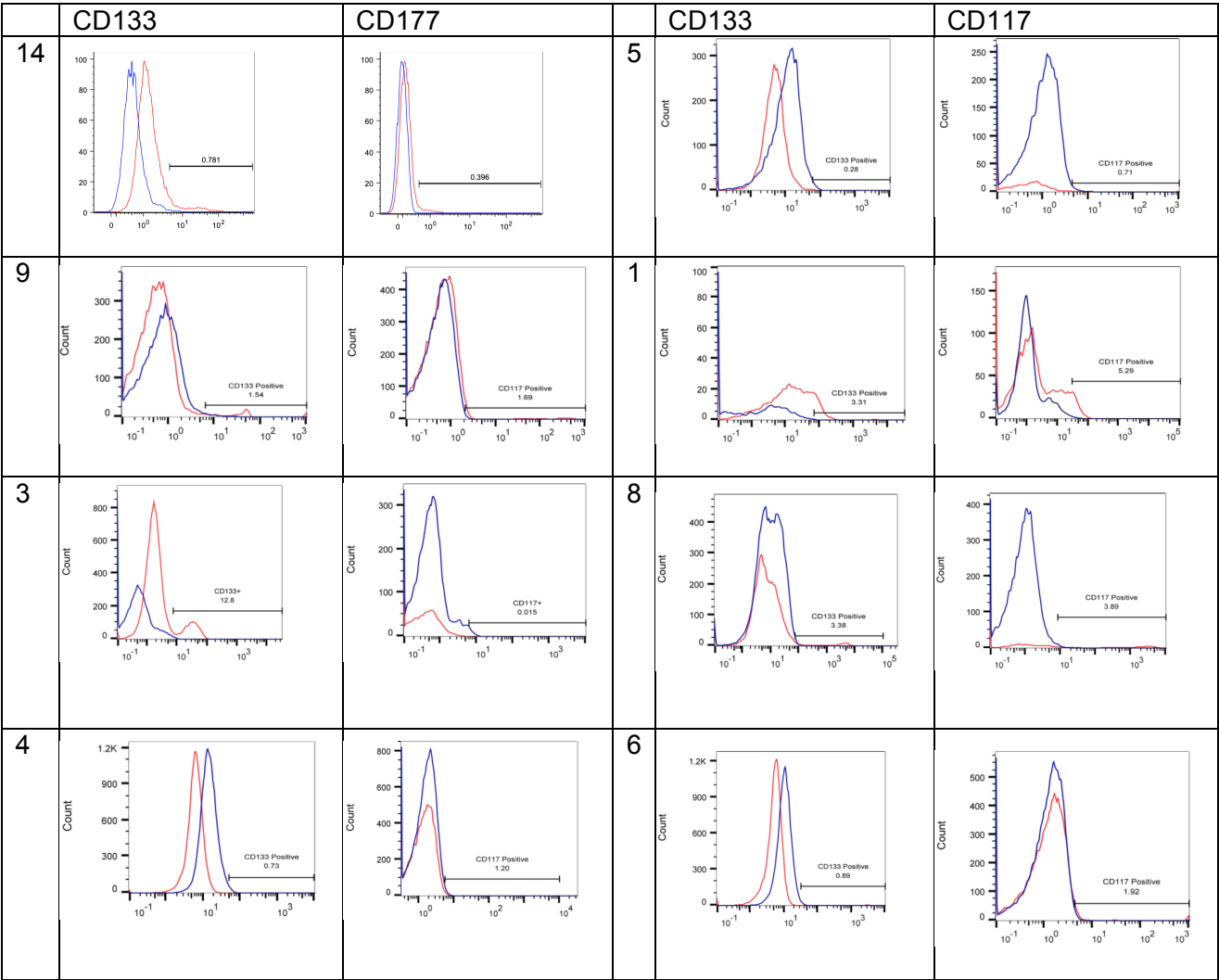

Supplement: Supplementary file 7 — Fig. S7. Flow cytometry histograms. In all panels, isotype is shown in red, antibody in blue. PDX numbers are shown to the left of histograms. Antibodies are as shown in column headings. [file MOL2-14-2796-s007.pdf]

## Supplementary Figure S8

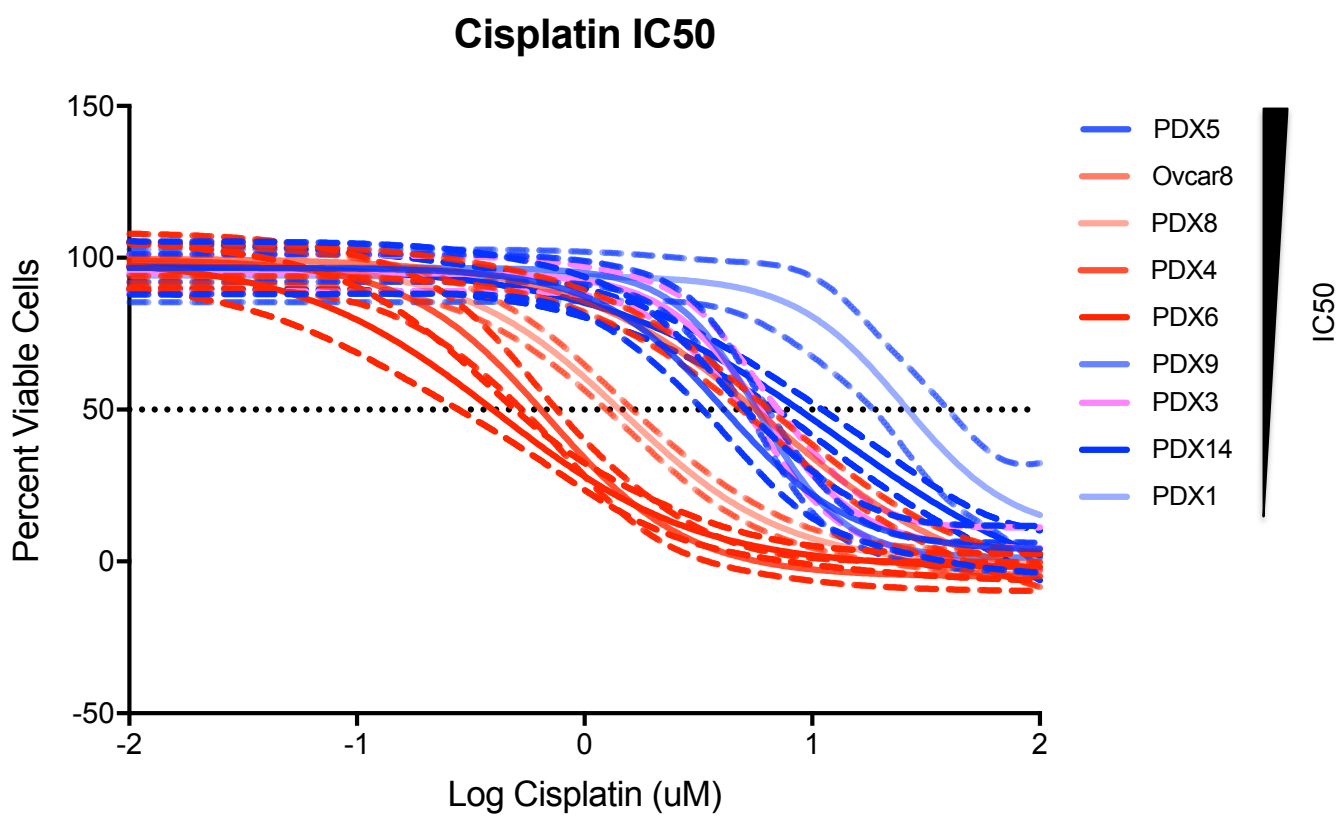

Supplement: Supplementary file 8 — Fig. S8. Cisplatin log curves demonstrate resistance in PD samples. Dashed lines represent 95% CI. Samples are arranged top to bottom in order of decreasing resistance. Independent biological replicates (n): PDX14 = 3, PDX5 = 7, PDX9 = 5, PDX1 = 3, PDX3 = 5, PDX8 = 4, OV8 = 4, PDX4 = 5, PDX6 = 5. [file MOL2-14-2796-s008.pdf]
