# Supplementary material for: Epithelial/mesenchymal heterogeneity of high‐grade serous ovarian carcinoma samples correlates with miRNA let‐7 levels and predicts tumor growth and metastasis
Source: Mol Oncol. 2020 Aug 21;14(11):2796–813. doi: 10.1002/1878-0261.12762 (PMC7607177; doi:10.1002/1878-0261.12762)
Supplement: Supplementary file 9 — Table S1. RT‐qPCR human primer sequences Table S2. Correlation between let‐7 levels and patient‐derived sample phenotypic and functional characteristics. [file MOL2-14-2796-s009.docx]

| Beta Actin F  Beta Actin R | TGAAGTGTGACGTGGACA TC  GGAGGAGCAATGATCTTGAT |
| --- | --- |
| Snail F  Snail R | GGCTGCTACAAGGCCAT  GCACTGGTACTTCTTGACATCT |
| E cadherin F  E cadherin R | TGCCCAGAAAATGAAAAAGG  GTGTATGTGGCAATGCGTTC |
| LIN28A F  LIN28A R | GAGCATGCAGAAGCGCAGATCAAA  TATGGCTGATGCTCTGGCAGAAGT |
| Nanog F  Nanog R | CAAAGGCAAACAACCCACTT  TCTGCTGGAGGCTGAGGTAT |
| HMGA2 F  HMGA2 R | AAAACGGCCAAGAGGCAGAC  ATGTCTCTTCAGTCTCCTGAGCA |
| OCLN F  OCLN R | TCAGGGAATATCCACCTATCACTTCAG  CATCAGCAGCAGCCATGTACTCTTCAC |
| TJP1 F  TJP1 R | AGGGGCAGTGGTGGTTTTCTGTTCTTTC  GCAGAGGTCAAAGTTCAAGGCTCAAGAGG |
| FN1 F  FN1 R | GCCTAAGCACTGGCACACAACAGTTT  ACTGCCATTGATGCACCATCCAAC |
| CLD3 F  CLD3 R | TTCATCGGCAGCAACATCATC  CGCCTGAAGGTCCTGTGG |

Supplementary Table 1. RT-qPCR human primer sequences

|  | ***Let-7a*** | ***Let-7b*** | ***Let-7d*** | ***Let-7e*** | ***Let-7g*** | ***Let-7i*** | **EMT score** |
| --- | --- | --- | --- | --- | --- | --- | --- |
| **Aspect ratio** | -0.85** | -0.8167* | -0.95*** | -0.9333*** | -0.9** | -0.8333** | -0.8871*** |
| **HMGA2 (Protein)** | -0.9667*** | -0.9333*** | -0.9** | -0.8833** | -0.8667** | -0.95*** | -0.7703* |
| **HMGA2 (RNA)** | -0.7636** | -0.7455* | -0.7182* | -0.7182* | -0.8273** | -0.7909** | -0.7505* |
| **LIN28A (Protein)** | -0.2556 | -0.1826 | -0.2556 | -0.2739 | -0.2556 | -0.2739 | -.07132* |
| **LIN28A (RNA)** | -0.463 | -0.4818 | -0.4545 | -0.4909 | -0.5 | -0.4727 | -0.6248 |
| **OCT4 (RNA)** | -0.2091 | -0.1636 | -0.2091 | -0.1818 | -0.1 | -0.1182 | 0.1126 |
| **NANOG (RNA)** | -0.1091 | -0.09091 | -0.1091 | -0.1 | -0.1273 | -0.03636 | -0.04826 |
| **Spheroid number** | -0.8356** | -0.8791** | -0.7921* | -0.7833* | -0.6 | -0.8269** | -0.4628 |
| **Spheroid size** | -0.8878** | -0.8965** | -0.8443** | -0.8182* | -0.705* | -0.8617** | -0.6224 |
| **Tumorigenicity** | -0.7407 | -0.9258* | -0.7407 | -0.8332 | -0.7407 | -0.8332 | -0.7707 |
| **End point** | -0.759 | -0.9411* | -0.759 | -0.8804* | -0.759 | -0.8804* | -0.7061 |
| **Tumor Burden** | -0.759 | -0.9411* | -0.759 | -0.8804* | -0.759 | -0.8804 | -0.7795 |
| **Metastasis** | -0.7549 | -0.4956 | -0.736 | -0.8181 | -0.7203 | -0.5734 | -0.5425 |
| **EMT Score** | 0.6535 | 0.5813 | 0.6458 | 0.6926* | 0.6765* | 0.6096 |  |
| **Migration** | 0.7167 | 0.7 | 0.6667 | 0.6667 | 0.4667 | 0.7167 | 0.7223* |
| **Cisplatin IC50** | 0.8667** | 0.8333** | 0.8167* | 0.8167* | 0.9167** | 0.8667** | 0.4804 |
| **Doubling Time** | 0.3833 | 0.4833 | 0.3833 | 0.4333 | 0.2833 | 0.4333 | 0.6244 |
| **CD133** | 0.5833 | 0.6167 | 0.4333 | 0.5 | 0.2333 | 0.65 | 0.4596 |
| **CD117** | 0.25 | 0.3 | 0.3167 | 0.3833 | 0.2 | 0.3167 | 0.2915 |
| **CD133/CD117** | 0.2667 | 0.2833 | 0.2167 | 0.25 | 0.1167 | 0.3 | 0.4816 |
| **Invasion** | 0.7143 | 0.6429 | 0.6071 | 0.5714 | 0.8214* | 0.6786 | -0.1931 |

**Supplementary Table 2.** Correlation between *let-7* levels and patient-derived sample phenotypic and functional characteristics. Spearman r values demonstrating negative correlation between *let-7* family members and pluripotency marker HMGA2, spheroid number and size, *in vivo* tumorigenicity, end point, tumor burden, metastasis, and EMT score. *: p ≤ 0.05, **: p ≤ 0.01, ***: p ≤ 0.001, ****: p≤0.0001.
